# Marine Applications of the Fast Marching Method

**DOI:** 10.3389/frobt.2020.00002

**Published:** 2020-01-28

**Authors:** Santiago Garrido, David Alvarez, Luis E. Moreno

**Affiliations:** Robotics Lab, Department of Systems and Automation Engineering, Universidad Carlos III de Madrid, Madrid, Spain

**Keywords:** fast marching, path planning, formations, vector field fast marching, trajectory planning

## Abstract

Path planning is general problem of mobile robots, which has special characteristics when applied to marine applications. In addition to avoid colliding with obstacles, in marine scenarios, environment conditions such as water currents or wind need to be taken into account in the path planning process. In this paper, several solutions based on the Fast Marching Method are proposed. The basic method focus on collision avoidance and optimal planning and, later on, using the same underlying method, the influence of marine currents in the optimal path planning is detailed. Finally, the application of these methods to consider marine robot formations is presented.

## 1. Introduction

Motion planning has been a very important field of research for many years. In the area of autonomous marine vehicles, both surface and underwater vehicles, some important aspects that are commonly optimized are travel time and safety conditions. This means that the path should avoid known obstacles and hazardous areas while reaching the goal pose as fast as possible.

An example of an approach using these concepts can be found in Bellingham and Willcox (1996), in which an underwater mission planning is proposed for optimizing energy consumption while guaranteeing spatio-temporal coverage. Following a similar goal, in Hert et al. (1996) the problem is formulated as a shortest path problem in order to guarantee the coverage of the terrain using a sonar system.

Besides, in marine environments, uncertainties due to the wind and water currents are complex and have a large impact on the path planning, as shown in Song et al. (2015). In order to deal with the environmental influence, a level set method based on the Fast Marching Method was proposed by Agarwal and Lermusiaux (2011). In Petres et al. (2005), an Anisotropic version of the Fast Marching Method (AFM) is used for submarine vehicles. This method provides collision free paths and their convergence is guaranteed, however, the water current model used does not take into account the power of the motor of the vehicle. Song et al. (2017) proposed an improvement of the AFM by using a multi-layered fast marching, which combines different environmental factors, such as currents and wind with attractive/repulsive maps. The proposed strategies deliver very interesting results, but do not guarantee the avoidance of local minima in the path planning due to the manner used to create the velocity maps.

The Fast Marching Method (*FMM*) and its evolution, known as the Fast Marching Square (*FM*^2^), have proven their value for path planning applications and robot motion because of their plasticity and ease of use. They have been applied to many different path planning related problems such as: indoors and outdoors (Garrido et al., 2017) robot motion, path learning (Gomez et al., 2017) or unmanned aerial (Álvarez et al., 2015) and marine vehicles (Petres et al., 2005; Song et al., 2017). However, all these methods are based on a scalar model of the environment. If vector fields are present in the model, then the Fast Marching Method subjected to a Vector Field (FMVF) is a better choice to perform the path planning.

In the next sections of this article, an overview of how the Fast Marching Method (FMM) works, as well as several path planning versions based on the FMM are explained. Besides, their basic characteristics and their use in marine-like environments are shown.

## 2. The Eikonal Equation and the Fast Marching Method

**Figure 1:**
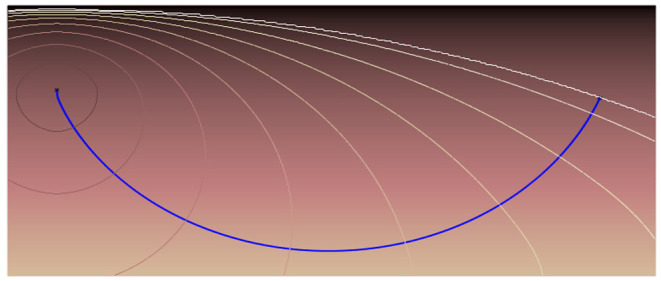
The resulting path of the light when the refractive index changes continuously.

The speed of light traversing different materials is defined as *v* = *c*/*n*, where *v* is the velocity in the specific medium, *c* = 300,000 *m*/*s* is the speed of light in vacuum and *n* is the refractive index which depends on the material that is traversed. For example, in water *n* = 1.33, while in glass *n* = 1.5, this difference provokes that when a ray of light passes from water to glass the ray changes its direction following the corresponding fastest path in each material. In cases in which there is a continuous change of refractive index, the path bends continuously, as in Figure 1. As it happens in a mirage in a hot road, the layers of air closest to the road are hotter than those that are further away. This creates a gradient of refractive indices that causes the rays coming from the sun to bend, therefore the driver has the optical illusion of seeing a kind of puddle of water on the road.

In general, the path that a ray of light follows (along any media) is the minimum in travel time. Therefore, the refractive index works as a viscosity or speed index that slows down the expansion of the light wave. Therefore, the path of a single ray of light among the wave expansion can be represented by its gradient.

One way to characterize the position of a front in expansion is to compute the arrival time, *T*, in which the front reaches each point of the space. For one dimension, the time of arrival value can be obtained simply considering that the traveled distance, *x*, is the product of the speed, *F*, and the time, *T*.

(1) $$\begin{array}{r} x=F\cdot T \end{array}$$

Then, the one dimensional spatial derivative of this function is:

(2) $$\begin{array}{r} \frac{dT\left(x\right)}{dx}=\frac{1}{F\left(x\right)} \end{array}$$

and therefore, the magnitude of the derivative of the arrival function *T*(*x*) is inversely proportional to the speed.

When considering multiple dimensions, the same concept is valid and the solution is found by substituting the derivative by the gradient, since the gradient is orthogonal to the level sets of the arrival time function *T*(*x*). In this way, the movement of the front of the wave can be characterized as the solution of a boundary condition problem. If the propagation speed depends only on the position, then equation 2 can be reformulated as the Eikonal equation:

(3) $$\begin{array}{r} |\nabla T\left(x\right)|F\left(x\right)=1. \end{array}$$

The Fast Marching Method (FMM) proposes a solution of the Eikonal equation for a grid map in which the velocity values at each point represent the refractive index. This artificial refractive index represents the cost function for the wave expansion. This method was originally proposed for a rectangular orthogonal mesh in Sethian (1996). As demonstrated in Yatziv et al. (2005), the FMM is an *O*(*n*) algorithm where *n* is the total number of grid points. The algorithm relies on an upwind finite difference approximation to the gradient as a first order solution of the differential equation.

The FMM is used for problems in which the speed function never changes of sign, which means that the wave front always moves forwards (no reflections are admitted). This characteristic allows to use a stationary formulation, because the wave front crosses each grid point only once. The wave propagation given by the FMM represents a distance function that corresponds to the Geodesic distance measured with the metric defined by the refraction matrix. This matrix indicates the speed of the wave front at each point of the grid.

### 2.1. Algorithm Implementation on an Orthogonal Mesh

In general, the FMM can model any phenomena which evolves as a wave front that propagates along its normal direction. Let *T*_*ij*_ be the time at which the wave front crosses the point (*i, j*) of a 2-dimensional map, satisfying |∇*T*|*F* = 1, the Eikonal equation. *F* represents the speed function and, therefore, *F* = *F*_*ij*_ represents the speed at each point of the map. As shown in Gómez et al. (2019), the most common first-order discretization of the Eikonal equation is given in Osher and Sethian (1988), which uses an upwind-difference scheme to approximate partial derivatives of *T*(**x**) (${D_{\text{ij}}}^{\pm x}$ represents the one-sided partial difference operator in direction ±*x*):

(4) $$\begin{array}{r} \begin{array}{c} T_{x}\left(\text{x}\right)\approx D_{\text{ij}}^{\pm x}T=\frac{T_{i\pm 1,j}-T_{\text{ij}}}{\pm \Delta_{\text{x}}}\\ T_{y}\left(\text{x}\right)\approx D_{\text{ij}}^{\pm y}T=\frac{T_{i,j\pm 1}-T_{\text{ij}}}{\pm \Delta_{\text{y}}} \end{array} \end{array}$$

A simple solution to Equation (4) is proposed in Sethian (1999):

(5) $$\begin{array}{r} \left\{\begin{array}{c} \max{\left(D_{\text{ij}}^{-x}T,-D_{\text{ij}}^{+x}T,0\right)}^{2}+\\ \max{\left(D_{\text{ij}}^{-y}T,-D_{\text{ij}}^{+y}T,0\right)}^{2} \end{array}\right\}=\frac{1}{F_{\text{ij}}^{2}} \end{array}$$

in which Δ*x* and Δ*y* are the grid spacing in the *x* and *y* directions. Substituting (4) in (5) and letting

(6) $$\begin{array}{l} T=T_{i,j}\\ T_{\text{x}}=\min\left(T_{i-1,j},T_{i+1,j}\right)\\ T_{\text{y}}=\min\left(T_{i,j-1},T_{i,j+1}\right) \end{array}$$

Then, for a discrete 2D space as, the Eikonal Equation can be written as:

(7) $$\begin{array}{r} \max{\left(\frac{T-T_{\text{x}}}{\Delta_{\text{x}}},0\right)}^{2}+\max{\left(\frac{T-T_{\text{y}}}{\Delta_{\text{y}}},0\right)}^{2}=\frac{1}{F_{\text{ij}}^{2}} \end{array}$$

Since the speed of the front is assumed to be positive (*F* > 0), *T* must be greater than *T*_x_ and *T*_y_ whenever the front wave has not already passed over the coordinates (*i, j*). Therefore, (7) can be simplified as:

(8) $$\begin{array}{r} {\left(\frac{T-T_{\text{x}}}{\Delta x}\right)}^{2}+{\left(\frac{T-T_{\text{y}}}{\Delta y}\right)}^{2}=\frac{1}{F_{\text{ij}}^{2}} \end{array}$$

Equation (8) is a regular quadratic equation of the form *aT*^2^ + *bT* + *c* = 0, where:

(9) $$\begin{array}{l} a=\Delta_{\text{x}}^{2}+\Delta_{\text{y}}^{2}\\ b=-2(\Delta_{\text{y}}^{2}T_{\text{x}}+\Delta_{\text{x}}^{2}T_{\text{y}})\\ c=\Delta_{\text{y}}^{2}T_{\text{x}}^{2}+\Delta_{\text{x}}^{2}T_{\text{y}}^{2}-\frac{\Delta_{\text{x}}^{2}\Delta_{\text{y}}^{2}}{F_{\text{ij}}^{2}} \end{array}$$

where, in order to simplify the notation, we assume that the grid is composed of unit square cells, that is, Δ_x_ = Δ_y_ = 1.

The full procedure to compute the solution of FMM is detailed in Algorithm 1. The algorithm classifies the points of the map into three sets: frozen, open and unvisited. Frozen points are those for which the arrival time cannot change anymore. Unvisited points are those that have not been processed yet. Finally, open points are those which can be considered as an interface between frozen and unvisited regions of the map, belonging to the propagating wave front.

**Algorithm 1:** Fast Marching Method

1: **procedure** FMM(*X*, *x*_0_)
**Require**: A grid map *X* of size *m* × *n*, source point *x*_0_.
*Initialization*.
2: **for all** *x* ∈ *X* **do**
3: *T*(*x*) ← ∞;
4: **end for**
5: *T*(*x*_0_) ← 0;
6: *frozen* ← *x*_0_;
7: $open\leftarrow N\left(x_{0}\right);$ ⊳ Neighbors of *x*_0_.
8: *open* ← *X*\(*frozen* ∪ *open*);
*Iteration*.
9: **while** *frozen* ≠ *X* **do**
10: $x_{1}\leftarrow \underset{x\in open}{\arg\min}d\left(x\right);$
11: **for all** ${\text{x}}_{\text{i}}=N\left(x_{1}\right)\in T\cap \notin frozen$ **do**
12: Update(**x**_i_);
13: *open* ← *open* ∪ {**x**_i_};
14: **end for**
15: *open* ← *open*\{*x*_1_}; ⊳ Updating sets.
16: *frozen* ← *frozen* ∪ {*x*_1_};
17: **end while**
18: **end procedure**

In the first step of the algorithm, the initialization, all the cells in the map are initialized with an infinite value (or the maximum value in the computing architecture) and set as unvisited, except for the starting point (the goal point in a path planning problem) which is set with an arrival time of 0 and considered as the first open point.

At each iteration, the open point with the smallest value of *T*(*x*) is set as frozen. Then, the arrival time of its von-Neumann neighbors is analyzed (if they are not labeled as frozen) by solving Equation (8). The value of a cell is updated if the computed arrival time is smaller than the actual one (*UPDATE* in Algorithm 1). This procedure continues until all points are set as frozen or the starting point of a path planning problem is reached.

**Figure 2:**
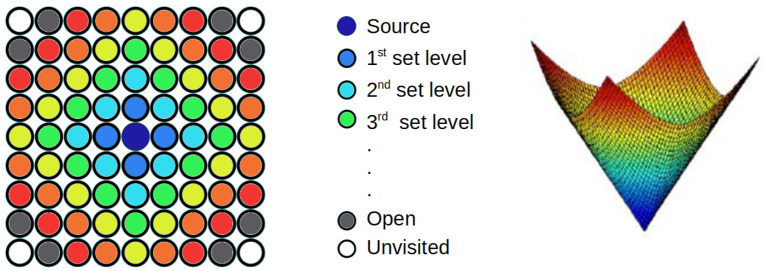
(Left) Wave propagation using the FMM. Different colors represent different arrival times. In gray, the points of the next iteration. In white, unvisited points. (Right) The final result represented using the time as a third axis.

Figure 2 shows the first steps of the algorithm, in which different colors are used to identify the different level sets. In the center, the dark blue point is the source of the wave. The gray points near the corners represent open points which will be solved in the next iterations, Finally, the white circles are unvisited areas. The computed arrival time function starts at the minimum value (*T* = 0) and grows toward larger values of *T*, forming a level-set solution with a unique global minimum. If the solution is shown using the time of arrival as the third axis, a funnel potential is formed, as it is appreciated in the right image of Figure 2.

**Figure 3:**
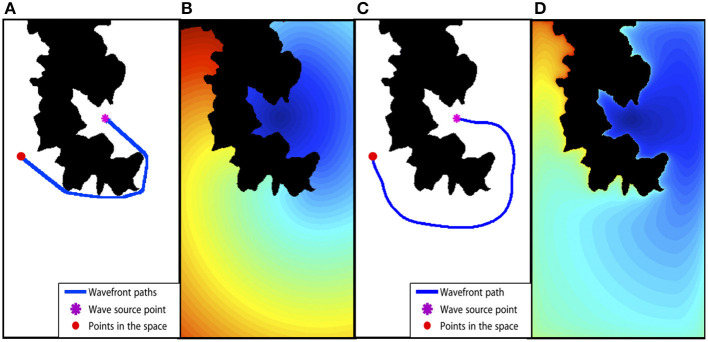
Fast Marching Method based path planning example. **(A)** The binary map used in the path planning and the path computed with FMM from start to end point. **(B)** The time of arrival map computed with FMM. **(C)** The resulting path using the *FM*^2^ method. **(D)** The time of arrival map obtained with *FM*^2^.

Finally, since the time of arrival function has a funnel-like shape, a vehicle's path toward its goal point can be extracted using the gradient descent method. Figure 3A shows an example of a path computed with FMM. Note that, although the path is optimal in time, it traverses the environment too close to the obstacles and, besides, forces the vehicle to perform abrupt turns. In Figure 3B, the resulting expansion of the wave based on the FMM can be appreciated. The different colors in the image indicate different arrival time sets, being the dark blue the smallest values while the red area corresponds to larger arrival time points. Note that, while the computed path is the shortest is distance and time of arrival, it is not a feasible path since the autonomous ship would need to travel too close to the coast, with a great danger of collision or run aground.

### 2.2. The Fast Marching Square Method

The Fast Marching Square Method (*FM*^2^) was introduced by Garrido et al. (2008) and consists on applying the basic FMM twice. Using this method, paths with an adequate smoothness and sufficient safety distances to the obstacles can be computed. The following procedure describes how the *FM*^2^ computes paths:

1. The environment is modeled in the same way as when using the FMM, a binary grid map (see Figure 3). The cells belonging to obstacles are labeled in black (a 0 value) and the cells corresponding to free space are labeled in white (a 1 value).
2. The first time the FMM is applied over the binary map, each cell labeled as an obstacle is used as wave source, expanding several waves at the same time. The resulting value of each cell in the map indicates the time the wave needs to reach the closest obstacle, therefore, it is proportional to the distance from obstacles since the wave moves at a constant speed in the whole map. Reversing the meaning of these values, they can be interpreted as the speed of the vehicle (and the speed of the wave expansion). This way, the resulting map is understood as the maximum admissible speed at each point of the environment, so that if the autonomous ship is near to obstacles, the admissible speed is lower than when is away from the obstacles. Finally, the speed values are rescaled to fix a maximum cell value of 1.
3. Then, the FMM is applied again over the environment. This time, the robot's goal point is used as wave source (a unique wave source to ensure one global minimum). The wave is expanded over the map until the initial point of the vehicle is reached. At each cell in the environment, the speed at which the wave expands is taken from the map computed in the previous step. It is important to keep in mind that this speed is lower the closer the vehicle (wave) is to obstacles. Figure 3D shows the time of arrival map resulting of this process.
4. Finally, gradient descent is applied over the time of arrival map from the starting point of the ship, and moving toward its goal point (the global minimum of the resulting map), obtaining the optimal path in terms of time of arrival, smoothness and safety, as shown in Figure 3C.

It is important to note that, when using this method in a real autonomous vehicle, the user must be aware of two critical aspects. First, the resolution used to model the environment where the robot moves. Since FMM is a grid based method, the higher resolution used, the better model of the environment and movement of the vehicle, at the cost of computational time, as shown in Gómez et al. (2019). Second, the user should consider the cells of value equal to 1 in the speed map as the maximum speed the vehicle is able to use (or the user wants to consider).

Next, some interesting modifications of the speed map, which allow to achieve different behaviors of the wave expansion (and therefore the computed paths) are going to be explained.

#### 2.2.1. The Flexibility of the Speed Map in *FM*^2^

Although the paths generated by the *FM*^2^ are good in terms of safety and smoothness, those paths can often be improved in terms of the traversed distance. For this reason, an adjustment parameter, α, that modifies the speed map to improve the planned path is proposed.

To perform the adjustment, each cell of the speed map, *F*_ij_, is raised to the power indicated by this parameter as in:

(10) $$\begin{array}{r} newF_{\text{ij}}=F_{\text{ij}}^{\alpha } \end{array}$$

When the value of α is lower than 1, the values in the speed map increase causing a lightening of the cells, which allows the wave expansion to use larger speeds. This causes the path to traverse the map closer to the obstacles. On the contrary, if the value is larger than 1, the cells are darkened causing paths stay further away from the obstacles.

Besides, it is commonly interesting to saturate the values in the speed map. For this reason, a value β is defined in the range of 0 and 1. The saturation is performed as follows: every cell in the speed map, *F*_ij_, with a greater value than β is set to one. Since the speed map is a distance function, this means that the wave moves at the maximum speed in all the cells in the map whose distance to the closest obstacle is greater than β. Therefore, the value of this parameter depends on the deceleration capabilities of the vehicles in use.

**Figure 4:**
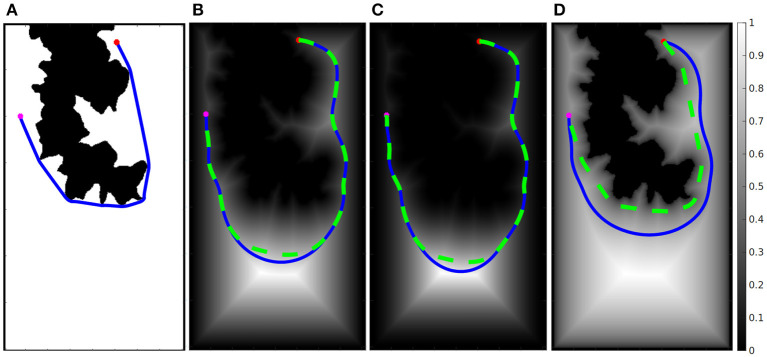
Different speed maps and paths obtained by modifying α- and β-values. In **(A)**, the original map is shown with the path computed using the basic FMM method. In **(B–D)**, the velocity maps computed with β = 1 for all images, and α = 1, α = 1.2, α = 0.4, respectively.

Figure 4 illustrates the effect of modifying α and β values. In Figure 4A, the original map is shown with the path computed using the basic FMM method, the start and end points are marked with a red and purple point, respectively. Figures 4B–D show the velocity map computed with β = 1 for all images, and α = 1, α = 1.2, α = 0.4, respectively. The resulting path is shown as a blue line. It is possible to appreciate that a value of α larger than 1 makes the velocity map to have greater values (darker in the image) which provoke the path to move farther from obstacles. On the other hand, when α is lower than 1, velocity values increase, allowing higher velocities around obstacles. Besides, for all cases, a second path is drawn using a dashed green line. This is the result of applying values β = 0.7, β = 0.8, β = 0.5, respectively. In all cases, the saturation value allows the path to move closer to obstacles, thus, reducing the path length at the cost of increasing the risk.

## 3. Fast Marching Method subjected to a Vector Field (FMVF)

The methodologies explained in the previous sections share a common key characteristic, in all cases the expansion of the wave deals with scalar speed values. However, there are situations in which a vector speed function may better reflect the environmental conditions in the path planning process. For example, in Garrido et al. (2016), a vector field is used to model outdoors characteristics interesting in mobile robotics, such as slopes or landslides.

In order to represent the movement of a ship in the water it is necessary to, not only take into account its direction, but also the effect of several vector variables such as wind flow or water currents. Mathematically, this can be done by computing a new cost function as in:

(11) $$\begin{array}{r} F_{ij}=F_{scal\text{\_}ij}+F_{vect\text{\_}ij} \end{array}$$

where *F*_*scal*_*ij*_ represents the influence of the scalar cost map and *F*_*vect*_*ij*_ represents the external vector fields. *F*_*vect*_*ij*_ is computed as the sum of external vector fields that affect the process. In the case of a ship, wind, tides and marine currents.

**Figure 5:**
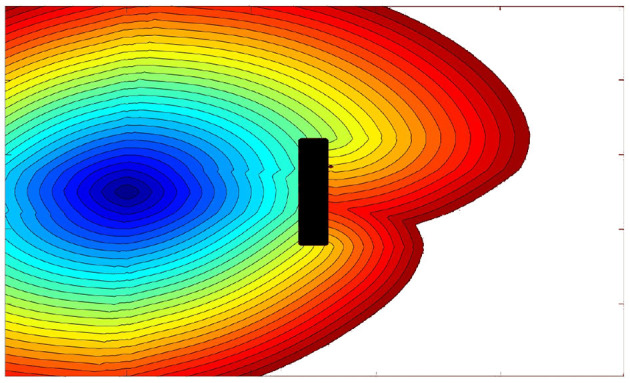
Fast marching expansion wave with a rectangular obstacle in the middle. The upper part of the environment is subjected to a unitary vector field pointing to the right. In the lower part, the field points to the left.

In Figure 5, the effect of an external vector field on a wave propagation calculated by the Fast Marching Method subjected to a Vector Field (FMVF) is shown. Note that there is a rectangular obstacle in the middle shown, in black color, where the wave collapses. It is easy to appreciate how the wave propagates faster in the area where the vector field points in the same direction as the expansion of the wave (upper part of the image).

It is important to note that the authors in Petres et al. (2005) and Petres et al. (2007) treated this subject previously. In these works, a normalization of the magnitude of the external vector field is performed without taking into account the magnitude of the scalar cost function. This makes a vector field with an intensity of 1 to have the same effect on the final path than one with an intensity of 10, minimizing the real influence of the external field. However, in this work, the function that is normalized is the total cost function:

(12) $$\begin{array}{r} \tilde{f}=f_{dif}+f_{vect} \end{array}$$

where *f*_*dif*_ is the cost function due to the distance to the obstacles in the environment converted into a vector field by:

(13) $$\begin{array}{r} f_{dif}=1-F_{ij} \end{array}$$

This way, the influence of the vector field over the velocity of the vehicle depends on their magnitude as well as on the angle between them, i.e., it depends on scalar product, and therefore the *f*_*vect*_ can be defined as:

(14) $$\begin{array}{r} f_{vect}\left(i,j\right)=1-\langle \nabla T_{i,j}\cdot {\vec{F}}_{i,j}\rangle \end{array}$$

Physically, this is equivalent to say that a force favors the ship when both external vector field and vehicle are pointing to the same direction.

It is very important to remind that the new cost function defined in Equation (12) must always be positive, because in the methods based on FMM the wave-front cannot move backwards. More details on the algorithm can be consulted in Petres et al. (2005, 2007).

**Figure 6:**
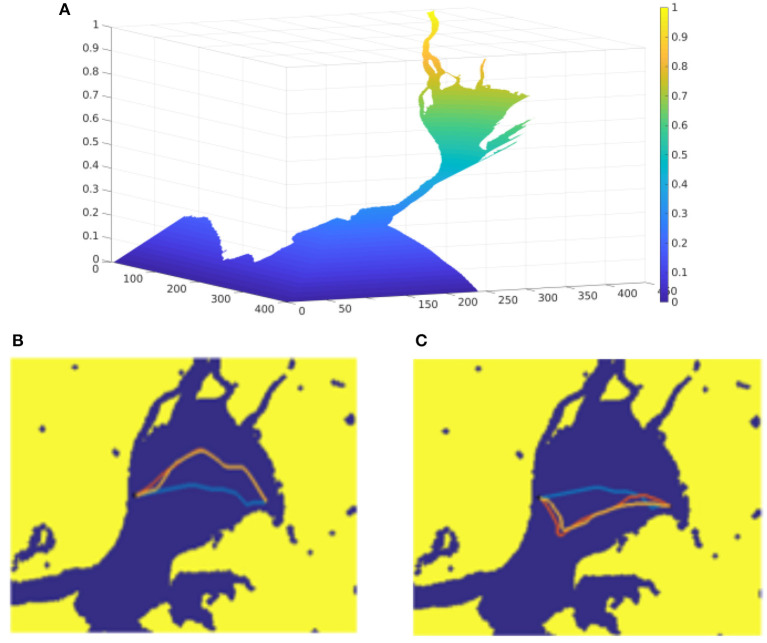
Comparison of the trajectories for different upwards and downwards tides. **(A)** Represents the function used to model the effect of tides in the estuary, which point toward the ocean. **(B,C)** Show different paths obtained with FMFV when tides point upwards, and downwards, respectively.

The next set of tests have been performed over a map of the Tagus River estuary, in the so-called Mar da Palha, in the city of Lisbon, Figure 6. In image A, the resultant wave expansion as a function of arrival time can be seen. Colors vary from dark blue for smaller to yellow for larger arrival time. This function is used to model the effect of tides in the estuary, which point toward the ocean (toward the dark blue area), inverting the sign makes the tides point in the opposite direction. In images B and C, different paths obtained with FMFV are shown. In Figure 6B the tides point upwards, while in Figure 6C tides point downwards, tides in both cases are identical in magnitude but in opposite direction. An example computed for a case in which the current is very close to zero, that is, the surface of the water is almost stationary, is shown in blue, which is used to analyze the influence of the introduction of a vector field of external forces. The magnitude of the currents is increased by a 5% from the yellow to the red test. It is clear that when the tide pushes either upwards or downwards, the calculated trajectories move away in comparison to the base trajectory (the blue one), since the vehicle undergoes a force that tends to take it away from the base path.

## 4. Path Following and Obstacle Avoidance Using Fast Marching Based Methods

In order to prove the smoothness of the paths computed with *FMM* based methods, a model of a real ship has been used to track them using a pure-pursuit method. The model uses a real-time control method for unmanned surface vehicles (USVs) based on Chaos et al. (2009).

**Figure 7:**
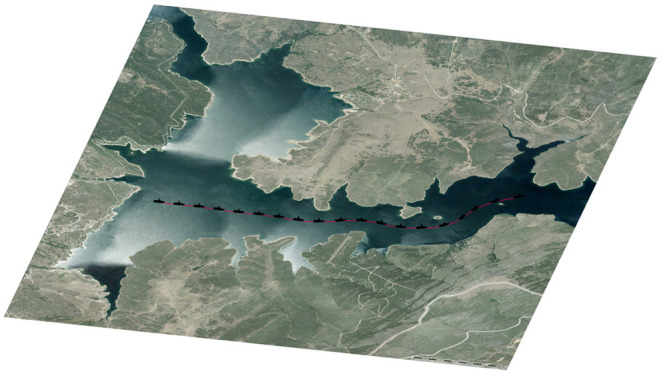
*FM*^2^ trajectory followed by the model of a USV.

Figure 7 shows the trajectory of a ship in the Atazar reservoir. Once the trajectory is computed with *FM*^2^, the path is followed by the model using the pure-pursuit method. The control loop uses the orientation error to compute the rudder angle that best follows the path, then, pure-pursuit is used to calculate the velocity of the ship taking into account the speed function of *FM*^2^. As shown in Figure 7, the calculated trajectory drawn in red, coincides with the poses of the ship reached using the simulated model.

In addition, it is commonly interesting for robots to be able to avoid obstacles while tracking their paths. Let us suppose that a mobile object is detected by the sensors in a ship (e.g., a LIDAR sensor) covering the path computed with *FM*^2^. Also, let us define a region of influence (*roi*) around the ship covering the path. This area indicates the space in which any obstacle can cause a collision. Using this information, the method uses a cyclic execution described in Algorithm 2. First, a path from the start to the end point is obtained using *FM*^2^. Then, pure pursuit is used to follow the path toward the next intermediate goal point. Next, if the end point is not reached, the *roi* is checked looking for mobile obstacles. When no mobile obstacle is detected, the path following continues with the original plan. However, when a mobile object is detected in this area, the previously computed path is no longer valid. In order to modify it, the velocity map is updated including the mobile object as a new obstacle, following the method explained in Garrido et al. (2013). The base of this update is to include a mobile obstacle location as an area with zero velocity (black in the velocity definition) which forces the wave expansion to avoid it. Since the velocity map is updated, a new path is computed (Second Potential). Therefore, the resulting new path avoids the mobile obstacle considering it as a static one during one control cycle.

**Algorithm 2:** Path Following and Obstacle Avoidance

**Figure 8:**
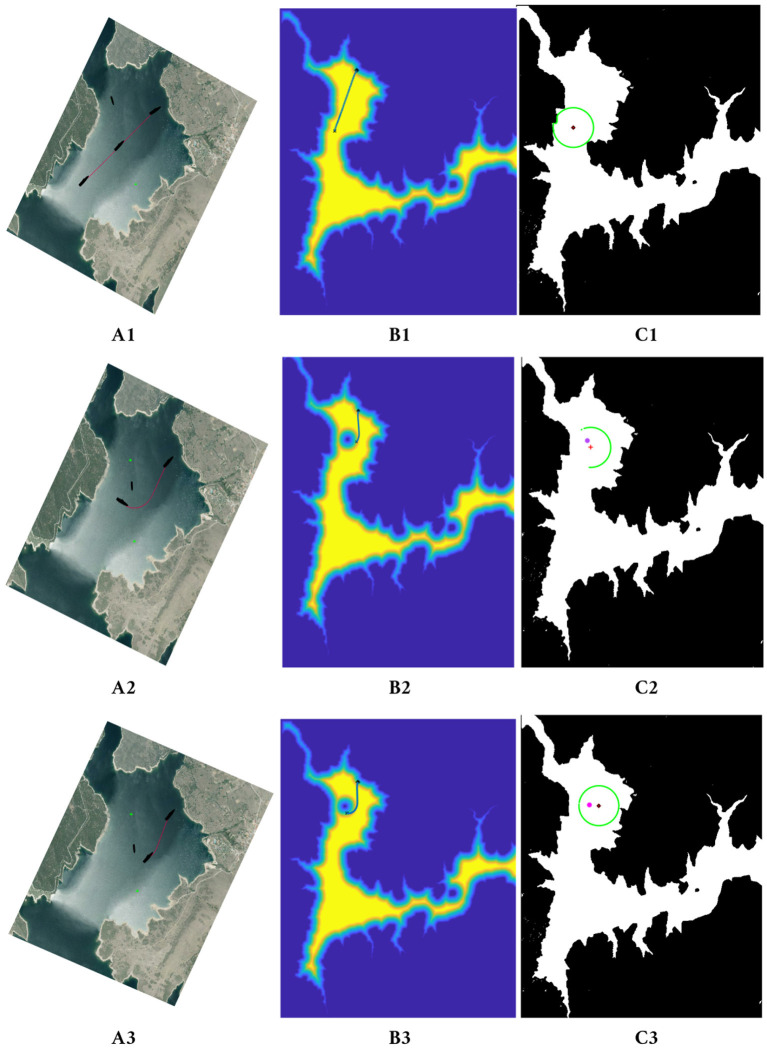
*FM*^2^ trajectory and path followed by the model of the ship avoiding the smaller ship moving from the left-upper corner. In **(A1–A3)** the sequence is shown on a satellite image of the reservoir. In **(B1–B3)** the mobile obstacle is included in the speed map as a circle. In **(C1–C3)** the ship following the path, its area of influence and the mobile obstacles are shown.

1: **procedure** Follow_path(*X*, *x*_*g*_, *x*_*a*_, *x*_*obs*_, *roi*)
**Require**: A grid binary map *X* of size *m* × *n*, goal point *x*_*g*_, robot actual position *x*_*a*_, location of obstacles at every iterations *x*_*obs*_, region of interest around the ship *roi*.
*First Potential*.
2: *vel* = *obtain*_*velocity*_*function*(*X*)
*Second Potential*.
3: $T=compute\text{\_}FM^{2}\left(x_{g},vel\right)$
4: *path* = *compute*_*path*(*x*_0_, *T*)
*Path Following*.
5: *rudder*_*angle* = *compute*_*angle*(*x*_0_, *path*)
6: *x*_*a*_ = *pure*_*pursuit*(*x*_*a*_, *rudder*_*angle, vel*)
7: **while** *x*_*a*_ ≠ *x*_*g*_ **do**
8: **if** *x*_*obs*_*a*+1_ ⊂ *roi* ∧ *x*_*obs*_*a*+1_ ≠ *x*_*obs*_*a*_ **then**
9: *vel* = *update*_*velocity*_*function*(*vel, x*_*obs*_*a*+1_)
10: **goto** *Second Potential*
11: **else**
12: **goto** *Path Following*
13:

Figure 8 shows a sequence (top to bottom) of how a ship avoids another ship that acts as a mobile obstacle interfering its trajectory. The different columns show the process using different maps. In column A, a satellite image of the Atazar reservoir is used to draw the ships and the path at each moment. In column B, the inclusion of the obstacle in the speed map is shown. The point where the obstacle is detected is modeled as an obstacle (dark blue in the example) and the allowed speed around this obstacle increases slowly, as happens around every static obstacle in the map. In column C, on the right, the ship which follows the computed path and its area of influence (a green circle) are shown.

In the first row of Figure 8, the original computed path taking into account only static obstacles is shown, together with the area of influence of the ship at the starting position. In the second row, the mobile obstacle is detected on the left side of the ship and, therefore, included as a new obstacle. Because of this change, the updated path avoids this area turning to the right. Finally, in the third row, although the obstacle is still in the area of influence, the ship can follow its path toward the goal safely.

## 5. Robot Formations

The algorithm described next is an extension of previous works. Firstly, in Garrido et al. (2013), the use of *FM*^2^ to control a robot formation in 2D environments was presented. Then, its usage with unmanned aerial vehicles (UAVs) was treated in Alvarez et al. (2014). In this section, its adaptation to marine-like environments will be explained.

The algorithm for controlling the robot formation is based on a leader-followers scheme. The leader can be a robot or even a virtual leader. Using the leader as a reference, the poses for the follower robots are defined by geometric equations to form the shape of the formation. Therefore, the goal poses of each follower along the path are a function of the leader's pose.

In the proposed solution, the path of the leader is computed without taking into account the other robots in the formation. This may cause the followers to move too close to obstacles or even collide with them. In order to avoid these situations, a shape deformation scheme based on the two-level artificial potential of *FM*^2^ can be used to calculate goal references to the followers during leader's navigation, as in reactive following. The main idea is to integrate an attracting potential toward the references of the formation (using the arrival time function) and a repelling potential from obstacles and other robots (the velocity/distances map).

**Figure 9:**
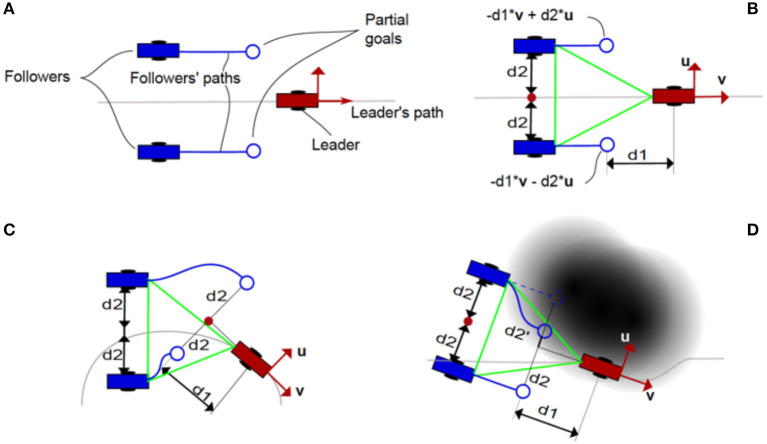
**(A)** Main components of the robot formation algorithm. **(B)** Reference geometric definition of a simple, triangle-shaped robot formation, note that the definition is based on vectors *u* and *v* (tangential and perpendicular to the path, respectively). **(C)** Behavior of the partial goals depending on the leader's pose. **(D)** Behavior of the partial goals depending on the obstacles in the environment.

Figure 9 shows an example of the use of the algorithm on a triangle-shaped robot formation. A 2D shape is used because it is easier to understand the behavior of the followers to avoid colliding with obstacles and among themselves. In Figure 9A, the main components of the robot formation are defined. In Figure 9B, the geometric definition of a triangle-shaped formation is presented, note that the tangential and perpendicular vectors of the leader's path are used as a reference. In Figure 9C, the goals of the followers adapt to the path of the leader's orientation. In Figure 9D, the use of the repelling potential to change the follower's partial goal and avoid obstacles in the environment is shown.

Algorithm 3 explains the integration of the control of the shape of the formation while covering the path. In the initialization (lines 2 to 7), the path for the leader is computed using *FM*^2^. Then, the formation covers the path in a control loop in which: first, the leader tracks its path (lines 9 and 10), then, new goal poses for all the followers are computed based on formation geometry (line 12) and closeness to obstacles (line 13). Finally, paths for the followers are calculated and tracked (lines 14 to 17). The control loop ends when the leader reaches its goal.

**Algorithm 3:** Robot Formation Control based on FMM

1: **procedure** Robot_Formation_Control(*X*, *x*_*g*_, *x*_*la*_, *x*_*obs*_)
**Require**: A grid binary map *X* of size *m* × *n*, goal point *x*_*lg*_ for the leader of the formation, leader actual position *x*_*la*_.
*First Potential*.
2: *vel*_*l*_ = *obtain*_*velocity*_*function*(*X*)
*Second Potential*.
3: $T_{l}=compute\text{\_}FM^{2}\left(x_{g},vel_{l}\right)$
4: *path*_*l*_ = *compute*_*path*(*x*_*la*_, *T*_*l*_)
5: **for all** k followers in formation **do**
6: *x*_*pg*_*k*_ = *formation*_*geometry*(*x*_*la*_, *vel*_*k*_)
7: **end for**
8: **while** *x*_*la*_ ≠ *x*_*lg*_ **do**
9: *rudder*_*angle*_*l* = *compute*_*angle*(*x*_*la*_, *path*_*l*_)
10: *x*_*la*_ = *pure*_*pursuit*(*x*_*la*_, *rudder*_*angle, vel*)
11: **for all** k followers in formation **do**
12: *x*_*pg*_*k*_ = *formation*_*geometry*(*x*_*la*_, *vel*_*k*_)
13: *x*_*pg*_*k*_ = *update*_*partial*_*goal*(*vel*_*k*_, *x*_*pg*_*k*_)
14: $T_{k}=compute\text{\_}FM^{2}\left(x_{pg\text{\_}k},vel_{k}\right)$
15: *path*_*k*_ = *compute*_*path*(*x*_*ka*_, *T*_*k*_)
16: *rudder*_*angle*_*k* = *compute*_*angle*(*x*_*ka*_, *path*_*k*_)
17: *x*_*ka*_ = *pure*_*pursuit*(*x*_*ka*_, *rudder*_*angle*_*k*_, *vel*_*k*_)
18: **end for**
19: **end while**

**Figure 10:**
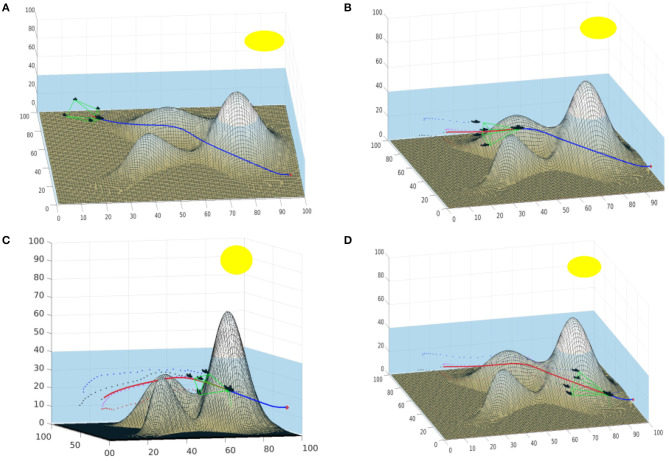
Example of a formation of submarines with a pyramid shape. **(A)** Shows the formation in the first steps of the movement. **(B,C)** Show the iterations when the formation traverses the area around the peaks. **(D)** Shows the formation approaching the goal point.

Figure 10 shows the use of this method in marine-like environments. The formation uses a pyramid shape with a squared base, the followers are located in the corners of the square. The leader and the followers are submarines. The numbers in the axis are related to the voxelization of the environment. In the default shape, the formation is oriented so that two submarines are located in the same vertical line (up and down) and the other two are located in the same horizontal line (right and left). The deformation function used allows each corner of the shape to shrink the base toward its center proportionally to the closeness to obstacles (as indicated in the velocity map). The maximum allowed deformation is a 70% of the total distance, to avoid collisions within the formation. The part of the path the leader has already covered is shown in red, while the part that is yet to be covered is shown in blue. The geometry of the formation is shown in green. The past poses of the follower robots are shown as small dots.

The path of the leader traverses the environment over the valley formed by two peaks. Figure 10A shows the formation in the firsts steps of the movement. Note that the follower moving close to the bottom of the sea shrinks its position correcting its height. In Figures 10B,C, when the formation approaches and traverses the area around the peaks, all the followers except the upper one need to shrink toward the center. These deformations are provoked because the velocity map in the areas the followers traverse have velocity values close to zero, indicating that an obstacle is near. Therefore, the square based in shrunk to increase the security of the path. In Figure 10D, the followers are farther from obstacles and therefore enlarge the base of the pyramid.

**Figure 11:**
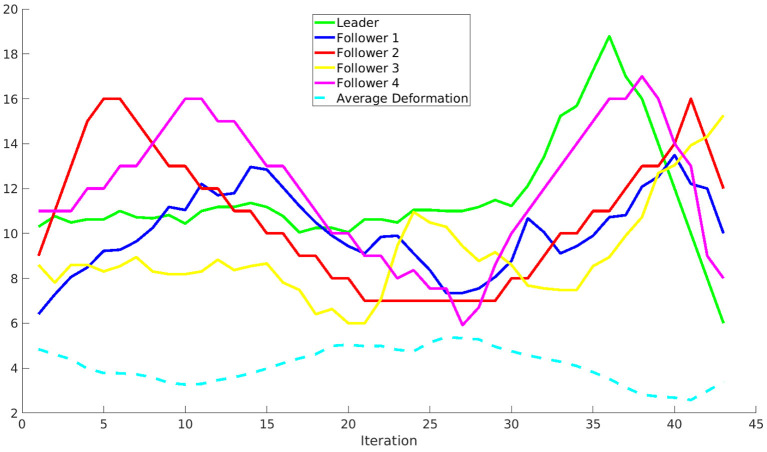
Distance of the leader and the followers to the closest obstacle in the environment at every step of the algorithm. Besides, the average deformation of the followers is shown as a dashed line.

Figure 11 shows the distance of the leader and the followers to the closest obstacle in the environment at every step of the algorithm. The distance is measured in voxels, so the real distance depends on the discretization used. Note how the distances are smaller in the central part of the path, in which the robots move between the peaks. Besides, the average deformation of the followers (also measured in voxels) is shown as a dashed line. Note that the deformation is larger when the distance to the obstacles is smaller.

## 6. Conclusions

In this paper, the use of the Fast Marching Method for marine-like environments is presented. Based on FMM, different versions of the wave expansion and path planning solutions are introduced, explaining the specific characteristics of each method and solutions, which may help a user to decide which FMM based method fits a particular application.

Besides, the usage of FMM based methods on real-time path following, obstacle avoidance and formation control are presented. On every section, simulated paths over digital environments are shown in order to appreciate the differences introduced by the proposed changes on the basic FMM. It is important to note that any of the explained FMM-like methods may be used to implement these applications. However, formation control has not yet been tested with the FMFV method, which is one the main future works.

Besides, future work will also focus on improving the models of the mobiles obstacles by using directional models and on extracting numerical results the safety provided by FMM-like path planning algorithms and the usage of robot formations in marine-like environments. Also, the implementation of these algorithms in a real autonomous marine vehicle is an important future work.

## Data Availability Statement

The datasets generated for this study are available on request to the corresponding author.

## Author Contributions

SG developed the FMVF method. DA performed the testing, wrote the algorithm explanations, and developed the Simulink model. LM participated on the discussion and the development of the techniques around FM2.

### Conflict of Interest

The authors declare that the research was conducted in the absence of any commercial or financial relationships that could be construed as a potential conflict of interest.
